# Performance of Humans vs. Exploration Algorithms on the Tower of London Test

**DOI:** 10.1371/journal.pone.0007263

**Published:** 2009-09-29

**Authors:** Eric Fimbel, Stéphane Lauzon, Constant Rainville

**Affiliations:** 1 Biorobotics Department, Fatronik Foundation, San Sebastian, Spain; 2 Ecole de technologie supérieure, Montréal, Quebec, Canada; 3 Institut universitaire de gériatrie de Montréal, Montréal, Quebec, Canada; 4 Centre de Recherche Interdisciplinaire en Réadaptation (CRIR), Centre de réadaptation Lucie-Bruneau, Montréal, Quebec, Canada; University of Vermont, United States of America

## Abstract

The Tower of London Test (TOL) used to assess executive functions was inspired in Artificial Intelligence tasks used to test problem-solving algorithms. In this study, we compare the performance of humans and of exploration algorithms. Instead of absolute execution times, we focus on how the execution time varies with the tasks and/or the number of moves. This approach used in Algorithmic Complexity provides a fair comparison between humans and computers, although humans are several orders of magnitude slower. On easy tasks (1 to 5 moves), healthy elderly persons performed like exploration algorithms using bounded memory resources, i.e., the execution time grew exponentially with the number of moves. This result was replicated with a group of healthy young participants. However, for difficult tasks (5 to 8 moves) the execution time of young participants did not increase significantly, whereas for exploration algorithms, the execution time keeps on increasing exponentially. A pre-and post-test control task showed a 25% improvement of visuo-motor skills but this was insufficient to explain this result. The findings suggest that naive participants used systematic exploration to solve the problem but under the effect of practice, they developed markedly more efficient strategies using the information acquired during the test.

## Introduction

The *Tower of London (TOL)* [1] was designed to assess deficits of planning in patients with lesions of the frontal lobe. In Shallice's rationale, these lesions damage the *Supervisory Attentional System (SAS*) responsible for the non-routine selection of *action schemes*. In the TOL, “the subject must construct a stack of objects from a starting configuration in series of individual moves” ([1], p. 203). Three beads placed on three rods are moved one by one in order to reach a given configuration. The subject performs twelve tasks requiring between 2 and 5 moves. With four moves or more, the SAS is presumably engaged, thus deficits are expected in patients with frontal lesions. Since then, the TOL has been used as a clinical tool, e.g., as part of the CANTAB (Cambridge Neuropsychological Test Automated Battery) computerized tests [2] and for research on executive functions and cognitive skills. For instance, the PubMed database contains 53 articles on the TOL (March 20, 2009; keywords “Tower London” and/or “TOL” in title; irrelevant references removed manually)

In the original TOL, the difficulty was graded by the number of moves. However, there is empirical evidence that the difficulty can vary markedly among the tasks with the same number of moves (see Section Discussion). It is now accepted that what really mediates the difficulty is the *search space* (also called *problem space*) [3], [4]. The search space is a graph that represents the possible configurations as nodes and the transformations (or moves) as edges. A task is defined by means of two nodes (initial and final configurations). A solution is a path of minimal length between these nodes. The search space allows determining the number of alternative paths, the configurations to examine, as well as several factors that may affect performance like the presence of conflictive moves or sub-goals [5]. Facts and figures on the search space of the TOL can be found at the web site that presents support information for this article [6].

The impact of the search space on the performance of problem-solving programs (*problem solvers*) has been known for long in Artificial Intelligence [7]. The search space determines the *combinatorial dimension*, i.e., the number of possibilities that problem solvers have to examine. The impact depends on the *algorithm*, i.e., the predetermined sequences of decisions and operations executed by the program. It also depends on the a priori information and on the memory resources. For instance a program with a priori information and no memory limitations can use a *look-up table* that contains a predetermined solution for each task. The solution is found in the table and the combinatorial dimension does not affect performance. Conversely, a program that has no memory and no specific exploration method will explore randomly the search space therefore the average execution time grows quickly with the combinatorial dimension.

It may seem straightforward to transpose explanations and results from problem solvers to human performance. In fact, Shallice [1] states explicitly that the architecture of the Supervisory Attentional System and the TOL itself were inspired from an earlier problem solver [8]. However, unless the contrary is proven, it would be premature to assume that human solve combinatorial problems like programs, i.e., by means of a predetermined *strategy* (in a broad sense, i.e., a way to solve a problem). It would also be premature to assume that human are systematic, i.e., that they employ the same strategy for all the tasks of a protocol. In fact, there is evidence of the contrary (see Section Discussion). Also, human performance may be affected by contextual and psychological factors (see Section Discussion). In summary, whether the search space affects in the same way programs and humans is unclear.

However, a simple approach issued from the field of Algorithmic Complexity allows comparing usefully human and program performance on a given search space. The objective is to determine the *degree of efficiency* of humans by placing the human performance curve (execution time as a function of the number of moves) on a discrete scale used to rate the efficiency of algorithms: constant, logarithmic, linear, polynomial, exponential… The numerical execution time of an algorithm is unimportant because it can be improved with faster computers. Conversely the pattern of variation is irreducible. For instance, algorithms with exponential patterns of variation are unusable for large-scale problems, whatever the computer.

In order to build a scale of efficiency, we select a few algorithms that solve the TOL efficiently in different conditions (a priori information or not, bounded vs. unbounded memory) and we determine their patterns of variation. We then perform correlation analysis. The pattern of variation with the highest correlation coefficient corresponds to the degree of efficiency of humans, whatever the method they employ to solve the problem. We can refine the method by using measures of performance of real algorithms (task by task) instead of patterns of variations (that only consider the number of moves).

In case of success, the approach will provide insights on the efficiency of human strategies and a fair comparison between humans and algorithms. Because the approach is based on correlation analysis, it works in spite of the facts that humans are several orders of magnitude slower, human strategies and their neuronal realizations are unknown, and human performance is affected by contextual and psychological factors of difficulty. Inasmuch as the correlations are strong, we may even use the patterns of variations as *algorithmic indexes* to predict quantitatively the degree of difficulty due to the search space. This would be a valuable outcome for experimental research using the TOL. Indeed, the approach may fail, for instance if human strategies are not constant during a test, if inter-individual differences are too important and/or if other factors of difficulty have more impact than the search space.

We nonetheless applied the approach to the data of healthy aged participants (from the study presented in [9]). Their performance fitted nicely with the algorithmic index of efficient algorithms using bounded memory resources and no a priori information. This result was promising but it was not considered a sufficient validation, among other reasons because the tasks were limited to 5 moves (like in the original TOL) whereas the search space allows tasks up to 8 moves. We thus conducted an experiment with healthy young volunteers. Because all participants had a high education level, we assumed that they were cognitively skilled and we included difficult tasks in the protocol.

## Materials and Methods

In the following, we refer to the original TOL [1]. We present the search space of the TOL and the algorithmic indexes, the experimental protocols for elderly and young participants and finally the data analyses.

### 1. Search space of the TOL

**Figure 1:**
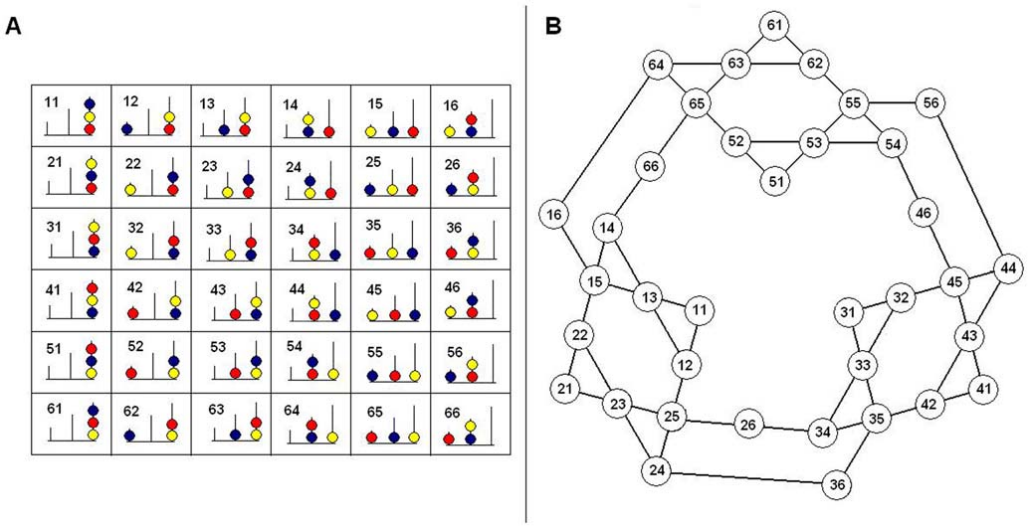
Search space of the TOL. *A*: configurations using nomenclature of [3]. *B*: search space. The nodes represent the configurations. The edges represent licit moves that transform a configuration into another (bi-directional).

The search space of the TOL (Figure 1) contains 36 configurations and 108 licit moves (i.e., 36 nodes and 54 bi-directional edges). The number of licit moves from a given configuration (*branching factor*) is 2, 3 or 4 (average = 3). The configurations present 6 *spatial patterns* and for each spatial pattern, there are 6 *color patterns* (i.e., the order in which the colors are painted on the balls). We use the nomenclature of [3] for the configurations and the patterns. There are 1296 possible tasks, requiring between 0 (trivial tasks) and 8 moves. The number of solutions per task range from 1 to 8. More information about the search space is available at [6]

### 2. Exploration algorithms and Algorithmic Indexes

The objective was to build a scale to which the strategies of participants can be compared. Each element of the scale corresponded to a class of algorithms that have the same pattern of variation. We represented this pattern by means of an *algorithmic index*, i.e., a curve giving the average execution time as a function of the number of moves (recall that the numerical values of the curve are unimportant, given that it will be used only for correlation analysis).

For practical reasons, we limited ourselves to a minimal scale composed of 3 indexes (see below). We assumed that it was sufficient to validate the method, and this limitation entailed no simplistic assumption on actual human strategies (the scale is only used to rate their efficiency; actual strategies may be quite different from the algorithms used to build the scale).

We considered *exploration algorithms*, capable of finding the shortest path between two configurations of the search space. Exploration algorithms are defined as follows.

- The algorithms receive as entry the search space of the TOL and the task to solve, i.e., a pair of configurations C_I_, C_F_ at a distance N
- They return a sequence of N moves between C_I_ and C_F_, i.e., a *shortest path*.
- They have no explicit information on the TOL, i.e., no predetermined data. Otherwise the problem could be solved in one step, with a look-up table.
- They embed no implicit information, i.e., they are not designed especially for the TOL. In other terms, they work with any (finite) search space.
- They are optimal given the constraints imposed to each family of algorithms, i.e., within each family, their pattern of variation has the slowest increase.

In other terms, the exploration algorithms are naive (like human participants that have not been exposed to the test) and they solve the general *problem of the shortest path*. We then computed four algorithmic indexes: U(N), B(N), I(task) and I(N). Their properties are summarized in Table 1. The numerical values of the indexes and the programs used to compute them can be found in the supporting web site [6].

**Table 1:** Algorithmic indexes.

index	description	pattern of variation
U(N)	unbounded memory, no a priori information	Linear, U(N)∼11N
B(N)	bounded memory, no a priori information	Exponential, B(N)∼e^1.15N^, i.e.3.16^N^
I(task)	bounded memory no a priori information, measured from a random algorithm	distribution around exponential, I(N)∼e^1.12N^, i.e.3.06^N^

*Pattern of variation*: indicates the trend line of the curve. Complete data available at [6].

*U(N): algorithms with unbounded memory.* These algorithms can store all the intermediate results. This speeds up the execution. Although unbounded memory is unrealistic for humans (this would be like using the long-term memory interactively) this family is of interest because it contains the most efficient algorithms for the general problem of the shortest path. The algorithmic index U(N) giving the average execution time T as a function of N increases as the number of nodes+arcs at distance N or lower. For the search space of the TOL, U(N) is almost linear. This can be attained for instance by means of *labeled broad-first exploration* [10]. Here is the sketch of such an algorithm
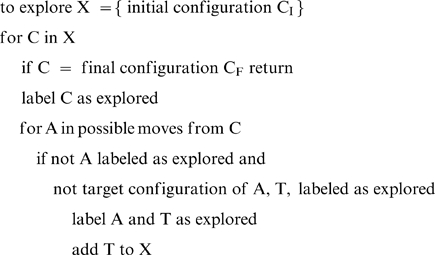



*B(N): algorithms with bounded memory.* These algorithms cannot store all the examined nodes and arcs because memory overflow may occur. These algorithms are therefore slower, but they are of interest because humans also have a bounded working memory [11], [12]. With bounded memory, it is at most possible to store the path under construction (here, 8 nodes or less). Because the paths have nodes and arcs in common, there is a considerable amount of repetition. B(N) increases as the number of paths of length N or lower, which in general grows as b^N^, b being the average branching factor [13]. This was verified for the search space of the TOL, i.e. B(N) grows exponentially with exponent close to 3 (Table 1). This can be attained for instance by means of depth-first exploration. Here is a sketch of depth-first algorithm.
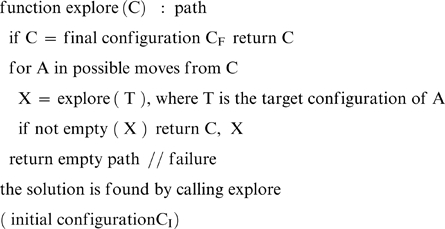



*I(task): task -specific index.* The indexes U(N) and T(N) do not discriminate among the tasks of N moves, whereas the combinatorial properties (and human performance) may differ markedly from task to task. We thus computed a task-dependent index I(task) that represents task by task the average execution time of efficient algorithms with bounded memory. To do so:

We implemented a *random broad-first exploration algorithm with bounded memory* that explores the nodes randomly in order of increasing distance from the initial configuration. The memory is used only to store the current path in order to avoid circuits (i.e., moving to a previous position. This requires at most 8 nodes), i.e., the algorithm uses bounded memory. Here is the sketch of the algorithm (complete algorithm can be found at [6]).
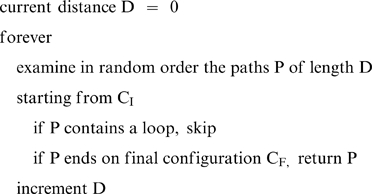

**Figure 2:**
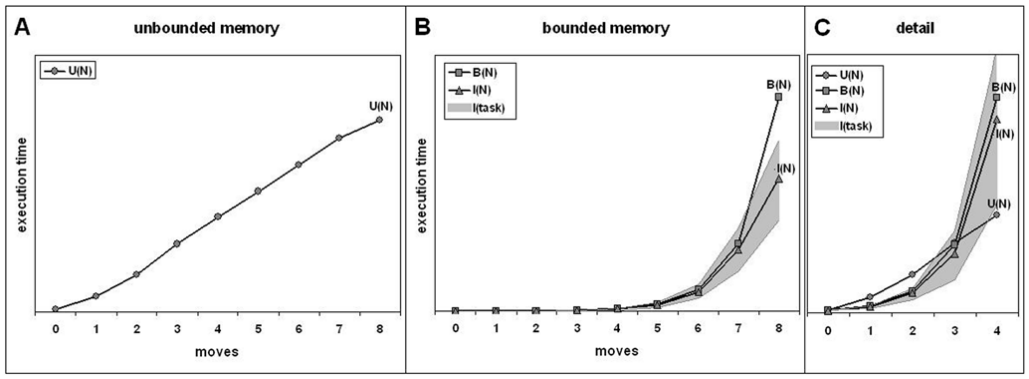
Algorithmic indexes. *Horizontal*: number of moves. *Vertical*: execution time (normalized). *A*: unbounded memory, U(N). *B*: bounded memory, B(N), I(task) (grey zone) and I(N), i.e., average of I(task), *C*: detail: all indicators together, N< = 4. According to Shallice (1982), with 4 moves or more, the supervisory attentional system is engaged.

I(task) was computed as the average number of steps (examined nodes + arcs) of the algorithm on 2048 repetitions. The number of steps is a robust indicator of the actual execution time (see *barometer instruction technique* [14]). Because at each repetition, the algorithm behaves differently, the average represents the performance of a collection of deterministic algorithms.
For validation, we computed I(N) as the average of I(task) for the tasks of N moves. We verified that I(N) was similar to B(N). This was expected, because the random algorithm uses bounded memory. Note that I(N) increases slightly slower than B(N), possibly because the algorithm does not examine the paths that contain loops (see exponents in Table 1, bottom right and Figure 2). However this small difference does not justify using I(N) as a separate algorithmic index.

### 3. Experiment 1 - elderly participants - protocol

The participants were tested in the context of a study presented in [9]. The group was composed of 35 healthy elderly volunteers randomly selected from a list of beneficiaries of a pension fund (14 males; age 72.4 σ = 4.4; education: 8.3 years, σ = 1.5). All of them were naive with the TOL and none presented history of cognitive and/or neurological diseases (exclusion criteria: stroke, Parkinson's disease, severe trauma with loss of consciousness for 48 h or more, depression and chronic alcoholism).

#### Ethic statement

All the participants gave written informed consent, according to the regulations of the Ethic Advisory Board of Université Bordeaux 2.

The TOL was presented under the form of two identical kits (initial and target configuration), made of a wooden base (22×6×2 cm) with 3 rods of 12 cm, 8 cm, 4.5 cm, and 3 balls (yellow, red and blue), 3 cm in diameter.

Two tasks of 2 moves were first executed by the examiner. The following instructions were then given to the participants: a) reproduce the target configuration in a minimum number of moves; b) move only one ball at a time; c) place at most one ball on the shortest peg, and two balls on the middle one; d) each ball can only move from one peg to another (i.e., do not lay the balls on the table or keep them in the hand). The participants were instructed to work out the minimal number of moves to reach the target configuration and to execute the corresponding sequence i) without errors and ii) as fast as possible. There were no time limits. They were asked to tell the examiner when they had finished, or when they abandoned. They performed 15 tasks presented in order of increasing number of moves, from 1 to 5 (Table 2). Each task corresponded to a unique trial. The number of moves was not indicated.

**Table 2:** Tasks and algorithmic indexes for elderly participants.

Task	Initial configuration	Final configuration	N	U(N)	B(N)	I(task)
1	12	13	1	7.00	3.00	2.00
2	53	52	1	7.00	3.00	3.00
3	23	24	1	7.00	3.00	3.00
4	12	15	2	17.33	9.67	9.00
5	23	36	2	17.33	9.67	11.00
6	53	65	2	17.33	9.67	11.00
7	23	35	3	31.67	30.67	36.00
8	53	64	3	31.67	30.67	37.00
9	12	16	3	31.67	30.67	29.00
10	53	16	4	44.33	97.67	118.00
11	12	64	4	44.33	97.67	94.00
12	23	33	4	44.33	97.67	92.00
13	12	63	5	56.17	310.67	275.00
14	53	15	5	56.17	310.67	362.00
15	23	32	5	56.17	310.67	293.00

The tasks are in the order of presentation. The configurations are identified according to Figure 1. *N*: number of moves.

The execution time (precision ±1 s) and the number of moves executed by the participant were measured by the examiner. The result (target configuration attained or not, abandon) was noted, as well as the rule violations that may have been committed.

### 4. Experiment 2 - young participants - protocol

Like in the original test [1], the tasks of Experiment 1 required 5 moves or less. In contrast, Experiment 2 was designed to cover more thoroughly the search space of the TOL, with tasks of 2 to 8 moves. This was presumably possible because the participants were young adults with high educational level, which were expectedly faster than the elderly participants of Experiment 1. Because the objective was *not* to compare the performance of young and elderly, it was of little interest to include the set of tasks of Experiment 1. The material (wooden kits, yellow, read and blue balls), the way of presentation and the instructions were similar to experiment 1.

The group was composed of 30 healthy young volunteers (13 males; age 22.9 σ = 3.2; education: 15.6 years, σ = 2.4). All of them were naive with the TOL. The exclusion criteria were: history of neurological diseases (like in Experiment 1), depression, motor deficits affecting hand movement, uncorrected vision or hearing deficits.

#### Ethic statement

All participants gave written informed consent according to the regulations of the Ethic Committee of IUGM (ethic certificate No. 20060101).

Participants executed a total of 35 tasks requiring between 2 and 8 moves (Table 3), in order of increasing number of moves, with 5 tasks per number of moves. Like in experiment 1, the number of moves was not indicated to the participant. The tasks of 1 move were not included because they were considered too easy. The tasks were selected pseudo-randomly so that the difficulty was balanced for each number of moves, i.e., the average of I(task) for the 5 tasks of N moves was close to I(N).

**Table 3:** Tasks and algorithmic indexes for young participants.

Task	Initial configuration	Final configuration	N	U(N)	B(N)	I(task)
1	23	12	2	17.33	9.67	11.00
2	21	15	2	17.33	9.67	7.00
3	14	22	2	17.33	9.67	9.00
4	26	12	2	17.33	9.67	6.00
5	56	43	2	17.33	9.67	6.00
6	33	44	3	31.67	30.67	36.00
7	34	43	3	31.67	30.67	29.00
8	53	63	3	31.67	30.67	28.00
9	63	53	3	31.67	30.67	28.00
10	64	14	3	31.67	30.67	23.00
11	46	34	4	44.33	97.67	67.00
12	22	65	4	44.33	97.67	86.00
13	33	23	4	44.33	97.67	91.00
14	22	34	4	44.33	97.67	95.00
15	65	11	4	44.33	97.67	117.00
16	22	62	5	56.17	310.67	303.00
17	31	23	5	56.17	310.67	183.00
18	52	32	5	56.17	310.67	302.00
19	44	26	5	56.17	310.67	230.00
20	32	23	5	56.17	310.67	276.00
21	21	31	6	68.67	988.67	591.00
22	32	11	6	68.67	988.67	970.00
23	23	45	6	68.67	988.67	924.00
24	22	31	6	68.67	988.67	881.00
25	41	12	6	68.67	988.67	654.00
26	14	31	7	81.00	3145.67	3050.00
27	25	53	7	81.00	3145.67	3840.00
28	36	65	7	81.00	3145.67	1932.00
29	46	15	7	81.00	3145.67	1929.00
30	63	31	7	81.00	3145.67	3678.00
31	22	46	8	89.50	10009.67	6542.00
32	24	51	8	89.50	10009.67	7324.00
33	26	62	8	89.50	10009.67	4260.00
34	35	65	8	89.50	10009.67	7271.00
35	56	21	8	89.50	10009.67	5053.00

The tasks are in the order of presentation. The configurations are identified according to Figure 1. *N*: number of moves. U(N), B(N), I(N): defined in Section Material and Methods.

During the test, the examiner recorded manually the total execution time (precision 1 s) and also the preparation time, i.e., the time elapsed between the presentation of the task and the first move. The difference represented the movement time. Note that on-line planning may occur during the movement time [15], [16]. In order to document the variation of visuo-motor performance (without planning demands), before and after the test, participants executed a sequence of 20 self-determined moves as fast as possible. The time was recorded manually by the examiner (precision 1 s). Note that the possible variation results from the opposite effects of fatigue and motor skill acquisition.

### 5. Data analysis

The execution time *T* was calculated for the *valid trials*, i.e., final configuration attained without rule violation whatever the number of moves.

The correlation coefficients (Pearson r) were computed on the set of tasks, between the averaged T (across participants) and the indexes U(N), B(N), and I(task). The indexes were then ranked in order of decreasing correlation coefficients.

For young participants, the correlations were initially computed for the whole set of tasks, but at the light of preliminary results, we computed them piecewise, i.e., on two subsets of tasks: *easy* (2 to 5 moves, n = 20) and *difficult* (5 to 8 moves, n = 20). The *easy subset* has a level of difficulty (or a number of moves) comparable to [1] and Experiment 1. The *difficult subset* contains the tasks of higher difficulty (or number of moves). The subsets are *not* disjoint (5 moves) so that both contain 20 tasks.

Within each set or subset of tasks, the significance of the differences between the correlation coefficients of the indexes was assessed by means of T-tests, using equation (1) [17].
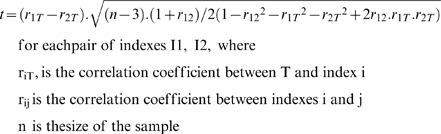
(1)


In order to determine whether inter-individual variability affect the ranking of the indexes, we also computed the correlation coefficients (Pearson r) between T (non-averaged, i.e., variable across participants) and the indexes U(N), B(N) and I(task) on the set of valid trials (Participants×Tasks).

Finally, for the young participants, in order to determine to what extent the variation of movement time may affect the total execution time (and the correlations), we compared the execution time of pre- an post-session visuo-motor tasks (20 self-determined moves) by means of a T-test.

## Results

### 1. Elderly participants - performance curve

**Figure 3:**
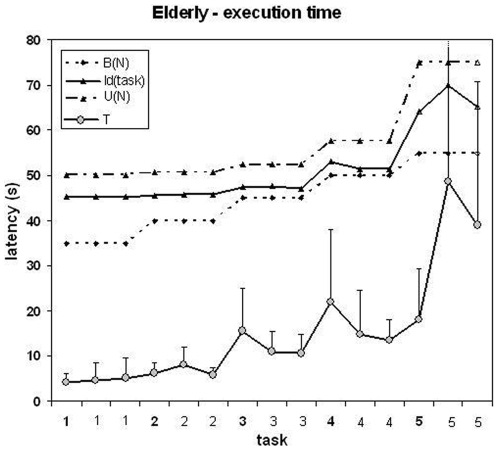
Elderly participants, execution time as a function of the task. *Horizontal*: tasks in the order of presentation. The scale indicates the number of moves. Vertical: latency (s). The indexes I(task), U(N) and B(N) are presented above (normalized amplitude, for clarity)

The execution time T as a function of the task is presented in Figure 3 with the algorithmic indexes. Two preliminary observations are of interest. 1) There were marked differences of average execution time between tasks with the same number of moves. The differences were so important that a task of 3 moves took on average longer than some tasks of 4 moves and the same occurred with some tasks of 4 and 5 moves. 2) There were a visual resemblance between the curves or T and I(task): both presented peaks (long execution times) for the same tasks of 4 and 5 moves. However, the foregoing observations are qualitative and cannot be generalized. In fact, given the high variability of execution times, we verified that some visually marked differences were not statistically significant.

The correlation coefficients (Pearson r) between the execution time and the algorithmic indexes are presented in Table 4. On the set of tasks (n = 15), I(task) presented the highest correlation coefficient (p = 0.92), followed by U(N) (0.86) and B(N) (0.81). All correlations were significant at p = 0.01. All the differences between correlation coefficients (as computed with Equation 1) were significant at p = 0.05. On the set of valid trials (n = 525) the correlation coefficients were lower because of the inter-individual variability. However all correlations were significant at p = 0.01. I(task) again presented the highest coefficient (p = 0.47) but the difference with B(N) (p = 0.46) was not significant. U(N) presented the lowest coefficient (p = 0.44).

**Table 4:** Elderly participants - correlation coefficients for the set of tasks and the set of valid trials.

	I(task)	U(N)	B(N)	Difference I-U	Difference I-B	Difference U-B
tasks n = 15	r = 0.92 **	r = 0.86 **	r = 0.81 **	t = 4.64 ***	t = 1.84 *	t>100***
valid trials n = 525	r = 0.47 **	r = 0.44 **	r = 0.46 **	t = 5.13 ***	t = 0.52 n.s.	t>100***

*Leftmost columns*: Pearson r between the execution time T and the algorithmic indexes. All correlations are significant at p = 0.01. *Bold*: best match. *Rightmost columns*: significance of the differences of correlation coefficients of pairs of indexes. t-values computed with Equation 1. Confidence levels: *: p = 0.05; **: p = 0.01; ***: p = 0.001.

In order to ensure that there was no better and simpler predictor of performance from the number of moves, we performed curve fitting for logarithmic, linear (like U(N)), polynomial, power law and exponential (like B(N) and I(task)) models. This confirmed that the best fit was for the exponential model (Pearson R of best fit: exponential: .94, power law: .90, 2nd order polynomial: .86, linear: .81, logarithmic: .73). Note that all the models have the same number of free parameters (2) except the polynomial (3). The exponential would therefore remain the best model for measures of quality of fit like the AIC or the Deviance

### 2. Young participants - performance curve

**Figure 4:**
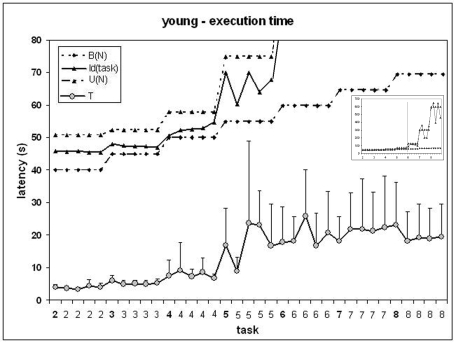
Young participants, execution time as a function of the task. *Horizontal*: tasks in the order of presentation. The scale indicates the number of moves. Vertical: latency (s). The indexes I(task), U(N) and B(N) are presented above. For clarity, the vertical scale is adjusted for tasks of 2 to 5 moves. *Snapshot* (upper right): indexes on the entire set of tasks.

**Figure 5:**
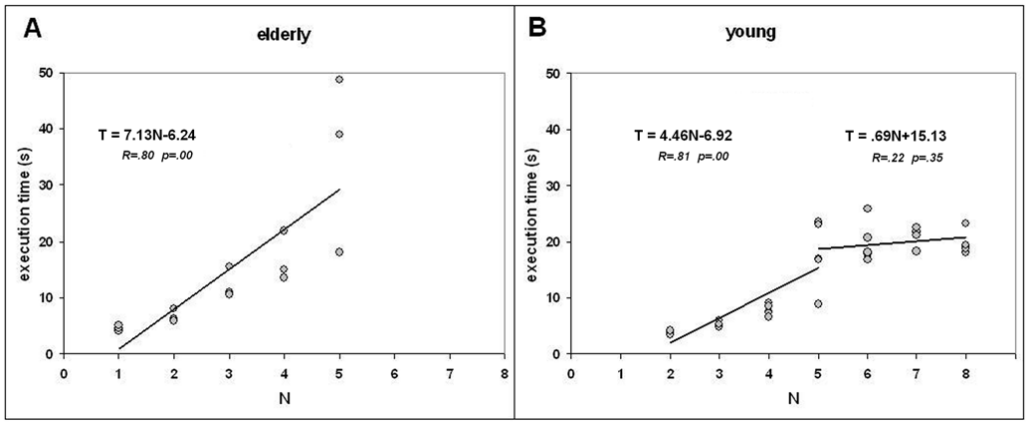
Slopes of the performance curves. *A*: elderly, *B*: young. Each dot represents a task. *Horizontal*: number of moves. *Vertical*: execution time(S). The trend lines and the equations are presented on the chart. For young participants, trend lines are determined separately for easy tasks (2–5 moves) and difficult tasks (5–8 moves).

The execution time T as a function of the task is presented in Figure 4. A visual examination provides the following preliminary observations. 1) Like for elderly participants, for easy tasks (2 to 5 moves) the execution time increased but 2) it presented no clear trend for the difficult tasks (5 to 8 moves). 3) The execution times were markedly shorter for young than elderly participants and the steepness of the curve for easy tasks was markedly lower. 4) Like for elderly participants, there were marked differences of execution time among tasks with the same number of moves. 5) There was a visual resemblance between the curves I(task) and T: both presented peaks (long execution times) for the same tasks of 5 moves. The differences of slopes and execution times are illustrated on Figure 5. However, the foregoing observations are qualitative and require a quantitative validation before any generalization (see below).

The correlation coefficients (Pearson r) between the execution time and the algorithmic indexes are presented in Table 5 separately for easy tasks (2 to 5 moves) (N = 20) and difficult tasks (5 to 8 moves). For easy tasks, I(task) presented the highest correlation coefficient (p = 0.92), followed by U(N) (0.90) and B(N) (0.80). All correlations were significant at p = 0.01. The differences between correlation coefficients (as computed with Equation 1) were not significant between I(task) and U(N), but significant at p = 0.001 between I(task) and B(N). On the set of valid trials (n = 596) the correlation coefficients were lower because of the inter-individual variability. However all correlations were significant at p = 0.01. I(task) again presented the highest coefficient (p = 0.56) but the difference with U(N) (p = 0.54) was not significant. The difference was significant with B(N) (p = 0.48) at p = 0.001.

**Table 5:** Young participants - correlation coefficients for the set of tasks and the set of valid trials.

	I(task)	U(N)	B(N)	Difference I-U	Difference I-B	difference U-B
2–5 moves						
tasks n = 20	r = 0.92 **	r = 0.90 **	r = 0.80 **	t = 1.24 n.s.	t = 2.74 **	t>100 ***
valid trials n = 596	r = 0.56 **	r = 0.54 **	r = 0.48 **	t = 1.60 n.s.	t = 4.39 ***	t>100 ***
5–8 moves						
tasks n = 20	r = 0.14 n.s.	r = 0.10 n.s.	r = 0.24 n.s.			
valid trials n = 597	r = 0.04 n.s.	r = 0.03 n.s.	r = 0.08 n.s.			

Results are presented separately for tasks of 2 to 5 moves and 5 to 8 moves. *Leftmost columns*: Pearson r between the execution time T and the algorithmic indexes. All correlations are significant at p = 0.01. *Bold*: best match. *Rightmost columns*: significance of the differences of correlation coefficients of pairs of indexes. t-values computed with Equation 1. Confidence levels: *: p = 0.05; **: p = 0.01; ***: p = 0.001.

As a validation, we performed curve fitting for logarithmic, linear, polynomial, power law and exponential models. This confirmed that the best fit was for the exponential model (Pearson R of best fit: exponential: .92, power law and 2nd order polynomial: .89, linear: .82, logarithmic: .76)

The results changed completely for the difficult tasks. None of the correlation was significant, whether on the set of tasks (n = 20) or the set of valid trials (n = 597). Note that this is a mere consequence of the flatness of the performance curve as depicted by Figure 5. It was thus pointless to compute the significance of the difference between correlation coefficients.

### 3. Young participants - preparation and movement time

**Figure 6:**
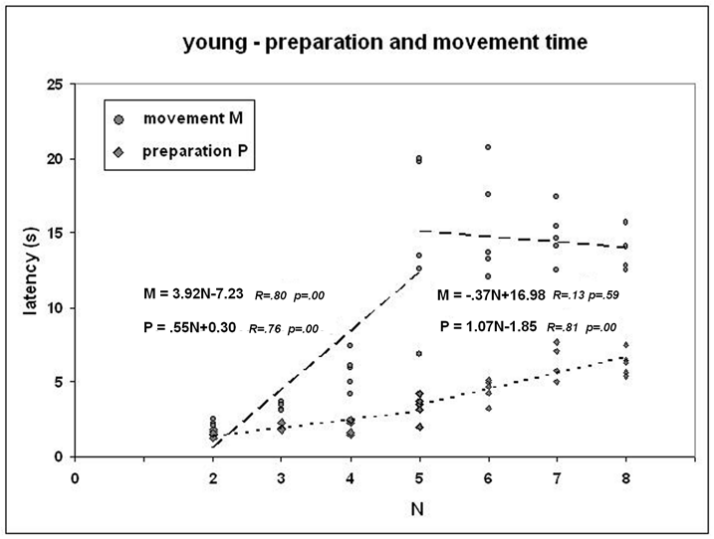
Preparation and movement times of young participants. Each dot represents a task. *Horizontal*: number of moves. *Vertical*: latency (averaged across participants). The trend lines and the equations are presented on the chart. The trend lines are determined separately for easy tasks (2–5 moves) and difficult tasks (5–8 moves).

In this section, we present minimal results on the preparation and movement times of young participants. The only objective is to provide cues to interpret the foregoing results because it has already been mentioned that there is on-line planning during the movement phase. Figure 6 depicts preparation and movement time as a function of the number of moves and the corresponding trend lines computed separately for easy tasks (2 to 5 moves) and difficult tasks (5 to 8 moves). For easy tasks, preparation and movement time increase with the number of moves. For difficult tasks, the preparation time increases but the movement time decreases slightly.

A t-test on the execution time of the pre- and post test visuo-motor tasks (execute 20 self-determined ball displacements as fast as possible) on the set of participants (n = 30) showed a significant decrease (pre: μ = 24.8 s σ = 7.1; post: μ = 19.8 s σ = 3.8; t = 5.7, significant at p = 0.01 bilateral). The amplitude of the decrease is about 5 s, i.e., 25%. In order to avoid misinterpretations of Figure 6, this decrease has to be contrasted with the 400% increase of the required ball movements (from 2 to 8) that tend to *increase* the total execution time.

## Discussion

### 1. Naive human strategies are as efficient as optimal exploration algorithms

For simple tasks (5 moves or less), humans and exploration algorithms with bounded memory had similar performances curves, i.e., the execution time increased exponentially with the number of moves. This result initially obtained with healthy elderly persons was conclusively replicated with young participants, on tasks of 5 moves and less.

The algorithms used as reference are optimally efficient under the same constraints than naive human participants: no a priori information, and bounded memory, similar to human working memory. These algorithms explore the configuration broad first, i.e., in order of increasing distance. However, this does not mean that humans use the same order. Any systematic exploration in which a node is examined a fixed number of times has a similar performance curve. In summary, the results support the view that naive humans use systematic exploration to solve the TOL.

We can reasonably discard that these results are artifacts. For easy tasks, the exponential indexes I(task) and B(N) were significantly more correlated to human execution time than the linear index U(N). Even when correlation coefficients were computed on the set of trials (accounting for the inter-individual variability), the index I(task) presented the highest correlation coefficient. This was verified for young and elderly, and on different sets of tasks.

### 2. Similar difficulty for naive humans and exploration algorithms

Due to the combinatorial properties of the search space of the TOL, the execution time may vary markedly across tasks with the same number of moves. This is true for humans, as depicted by performance curves (Figures 3 and 4) and for exploration algorithms as shown by the numerical values of the index I(task) that characterizes the average execution time of exploration algorithms on each task [6].

In addition, the results support the view that naive human strategies and exploration algorithms are similarly affected by the combinatorial properties of the search space. In both experiments, the specific index I(task) (r = 0.92) had a significantly higher correlation coefficient than the general indexes B(N) and U(N). Note that I(N), the task-independent version of I(task) is similar to B(N), and its correlation coefficients would have been similar, i.e., significantly lower than those of I(task). This shows conclusively that there is a significant benefit in using a task-dependent algorithmic index.

The results also show conclusively that the number of moves N is a poor predictor of human performance, at least in comparison with I(task). Note that the index U(N) (that corresponds to algorithms with unbounded memory) is almost a linear function of the number of moves. Thus N would have obtained correlation coefficients similar to those of U(N) (about 0.80 for both groups), significantly lower than those of I(task).

This suggests that practitioners and researchers working with the TOL could beneficially use I(task) instead of N in order to grade the difficulty of the tasks. This index can be found at [6]. I(task) represents the *combinatorial difficulty*, i.e., the difficulty due to the configuration of the search space, which is constant whatever the features of the participants, the protocol and the environment. However, it is worth underlining that I(task), like the number of moves is only a coarse predictor of difficulty. It cannot account for the variety of factors that may affect human performance.

Some of these factors can be obtained from the search space, like the presence of positive or negative triggers (i.e., initial moves that place a ball immediately in its final position; triggers tend to be intuitive moves for naive participants, but only positive triggers lead to some solution) [9] or the presence of conflictive moves or sub-goals [4], [5]. Other factors are related to the protocol, like the physical model, i.e., the nature of objects and actions used to present the task [18], the instructions [16], [19], the way of presentation, computer screen vs. real objects [20] or the presence of prior information [19]. Finally, some factors of performance are external to the task and the protocol, e.g., mood [21].

### 3. Non-naive human strategies more efficient than exploration algorithms

The surprising result occurred during the second half of the session of young participants: their execution time did not increase although they had to solve tasks of increasing difficulty (as graded by the number of moves). This means that humans became markedly more efficient than the exploration algorithms that best described their naive performance. It is unlikely that this result is an artifact. All the correlations between human execution time and algorithmic indexes that were significant during the first half of the test became non-significant on tasks of 5 moves and more, as an effect of the flatness of the performance curve (Figure 5).

The change of efficiency is in line with the general literature on automaticity [22], [23], [24] and skills acquisition [25], [26]. It is admitted that general intelligence (and/or controlled execution and/or executive functions) is employed to execute a novel task. Conscious control and attention are required, and the execution is slow, sequential and effortful. With practice, the execution requires less attention, less conscious awareness, and becomes more efficient. However, the gain in efficiency may come from a shift towards ‘expert’ strategies (in line with the Principle of Rationality, [27]) and/or a faster execution of the basic operations while strategies remain unchanged.

In the present case, it is unlikely that the strategies remained identical while basic operations became more efficient (e.g., visual check and mental representation of configurations, mental rehearsal of moves, physical movements). If this was the case, the execution time would have decreased during the sequences of 5 tasks with the same number of moves, and this did not occur (Figure 4). Also, the performance at the pre- and post-test visuo-motor control task only increased about 25%, but this is unlikely to compensate for the increase of the number of ball movements, i.e., 400% on the whole test (2 to 8 moves) and 60% on the second half (from 5 to 8 moves). Although the number of required movements only determines indirectly the visuo-motor demands, we may expect that such demands increased more than 25%.

The change in efficiency is more likely due to a change of strategies. This explanation is in line with evidence obtained from changes in brain activation related to cognitive skill acquisition [28]. It is also in line with evidence obtained by the patterns of gaze [29] that suggests that the difference between good and bad performers corresponds to a difference in strategy (although it may also come from a more thorough planning, [30]).

Indirect evidence from the algorithmic indexes provides additional insights about the strategies employed in the second half of the test. The algorithms used as reference are optimal given the conditions of no a priori information and bounded memory. The only possibility to improve efficiency is thus to release these constraints, and in the case of humans, the most likely explanation is the presence of a priori information. Non-naive participants may collect information (in a broad sense) during the test and employ it to improve their efficiency. If this is the case, the new theoretical limit is settled by algorithms with total a priori information and no memory limitations (e.g., using a look-up table that contains the solutions). In the best case, trained humans could solve the TOL in (almost) constant time whatever the task.

### 4. What are the non-naive strategies?

The type of information acquired during the test and the differences naive and non-naive strategies remain open issues. The evidence from [29], [30] is about good vs. bad performers. In a case study on the tower of Hanoi (TOH), information on the strategies was obtained from observation and verbalization [31]. However, the search spaces of the TOL and the TOH are markedly different. In addition, the TOH can be solved by applying systematically the same sequence of operations [32]. This recursive algorithm is often used as example in Computer Science, e.g., [14]. Such a systematic method does not exist for the TOL.

From the verbalizations of TOL (students that used the TOL in academic projects at ETS, 2001 to 2003), we may hypothesize that some of them recognize intermediate sub-goals and use stereotyped subsequences to attain them, like permuting two balls on the same peg (“inversion”) or moving a stack of balls from a peg to another (“translation”). This was also informally observed by the authors in different experiments. After the “inversion method” (which requires four moves) was discovered by a participant, posterior inversions were rapidly identified and executed. *Chunking* (i.e., the decomposition of the task in intermediate sub-goals and sub-sequences) reduces the number of intermediate configurations to examine and increases efficiency.

An indirect evidence of chunking is obtained from the data on preparation vs. movement time. The movement time results from overlapped visuo-motor activity and on-line planning [15], [16]. On-line planning is a manifestation of chunking, i.e., intermediate sub-goals are solved on-line, not during the preparation phase. The present data, i.e., small increase of preparation time with the number of moves and movement time almost independent of the number of moves in the second half of the test, are compatible with the presence of chunking: the number of sub-goals increase moderately with the number of moves (preparation time to determine sub-goals), and the sub-goals are attained by means of stereotyped sequences (no need to plan them completely, which may explain why the on-line planning time does not increase exponentially). However, as said before, these evidences are only indirect and require further research.

### 5. Against over-generalization and over-interpretation

The reader should be warned against *over-generalization* of the foregoing results. For historical reasons, the TOL is a common playground for humans and algorithms. To humans, the TOL offers a familiar physical model (balls and pegs) and a reasonable gradation of difficulty. To algorithms, the TOL offers a crown-shaped search space that allows efficient algorithms to make the difference. Although the present results may be of interest for researchers and practitioners using the TOL, from a theoretical viewpoint, they are only illustrative, i.e., they document a case where human strategies are more efficient than optimal algorithms.

A second risk is *over-interpretation*, i.e., to extend the analogy between humans and computers beyond the fact that they use similar basic operations to solve the TOL, i.e., examine configurations and moves, build a sequence of moves. Brain-Computer and Mind-Computer analogies are pervasive, whether computers are used as metaphors to explain the brain [33], as a tool to reproduce brain functions [34] or simply as a source of explanation schemes, e.g., “programs”, “functional blocks”, “memory registers”, “parallelism”, “networks”. Here, we only compared the *performance* of humans and algorithms. In fact, the present results are a clear warning against unfounded computer metaphors.

### 6 Concluding remarks

The present study was initiated after a discussion in which two of the authors shared their experiences on the TOL. One of the authors (CR, neuropsychologist) focused on the psychological factors and the deficits that affect human performance. The other (EF, computer scientist) focused on the search space and the algorithmic complexity. It became clear that both perspectives were useful but not self-sufficient. Administering a TOL test without considering the combinatorial difficulty and the search space may lead to misinterpretations. Conversely, reducing the tasks to an exploration of the search space is at best simplistic.

During the gestation of this study, several articles established relationships between computational and/or combinatorial aspects of the TOL and the difficulty of the tasks, e.g., [3], [4], [5]. The contribution of the present study is a new method to compare human strategies and algorithms on the basis of their efficiency. Although this contribution may be modest, it provides at least a simple way to compare humans and computers without using simplistic analogies.
